# Supplementary material for: Large-Area Biomolecule Nanopatterns on Diblock Copolymer Surfaces for Cell Adhesion Studies
Source: Nanomaterials (Basel). 2019 Apr 9;9(4):579. doi: 10.3390/nano9040579 (PMC6523780; doi:10.3390/nano9040579)
Supplement: Supplementary file 1 [file nanomaterials-09-00579-s001.pdf]

nanomaterials

Supplementary Materials

The following are available online at www.mdpi.com/xxx/s1.

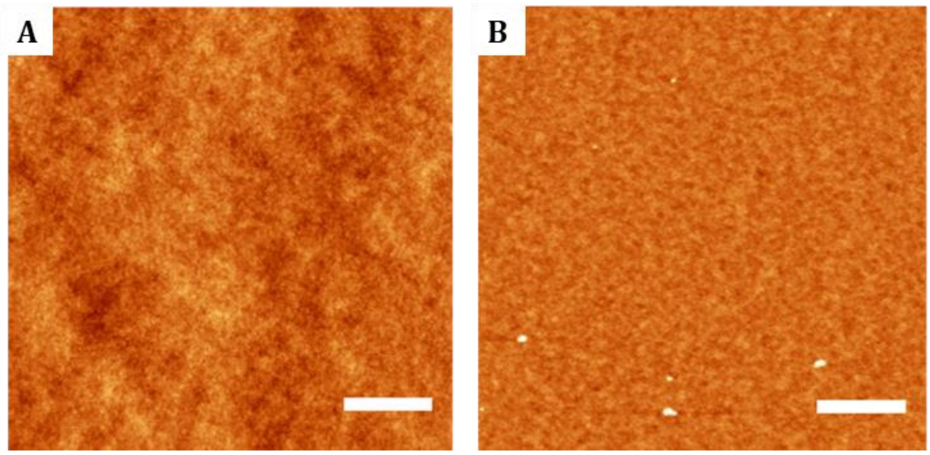

Figure S1. AFM topographical images of thin films of PS-r-PMMA-α-hydroxyl- -tempo (molecular weight 15500 kg/mol, polystyrene fraction 0.71) formed by spin coating at 2.5 mg/mL. (A) As deposited. (B) Resulting polymer brush layers after washing with fresh toluene. (Scale bar: 400 nm; Z-scale: 5 nm).

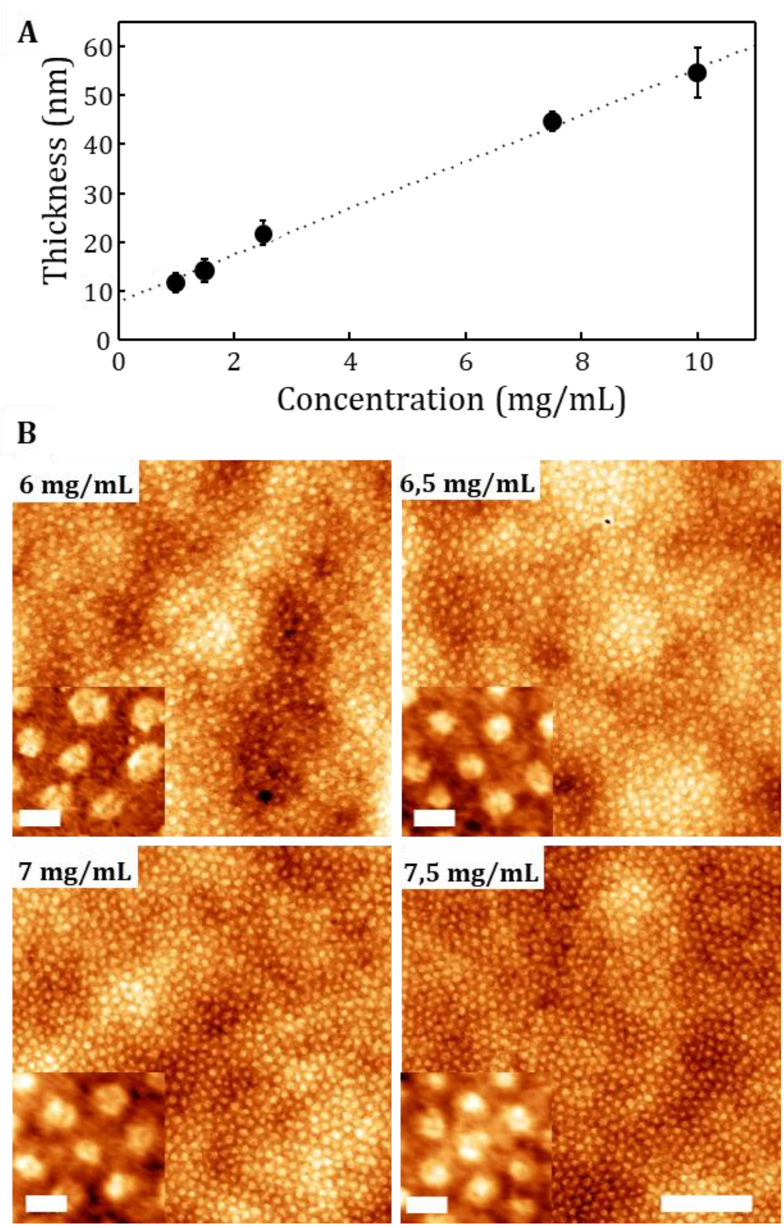

Figure S2. (A) Film thickness dependence on block copolymer concentration. Plotted values correspond to PS-b-PMMA 123-35 diblock copolymer spun-coated at 3000 rpm for 40 s. Values followed a linear fitting (R^2=0.9951). (B) Topographic AFM images of PS-b-PMMA 123-35 diblock copolymer spun-coated at 6 mg/mL, 6.5 mg/mL, 7 mg/m, and 7.5 mg/mL (Scale bar: 400 nm; Z-scale: 5 nm). Insets show a representative zoom of the hexagonal arrangement for each polymer concentration (Inset scale bar: 38 nm).

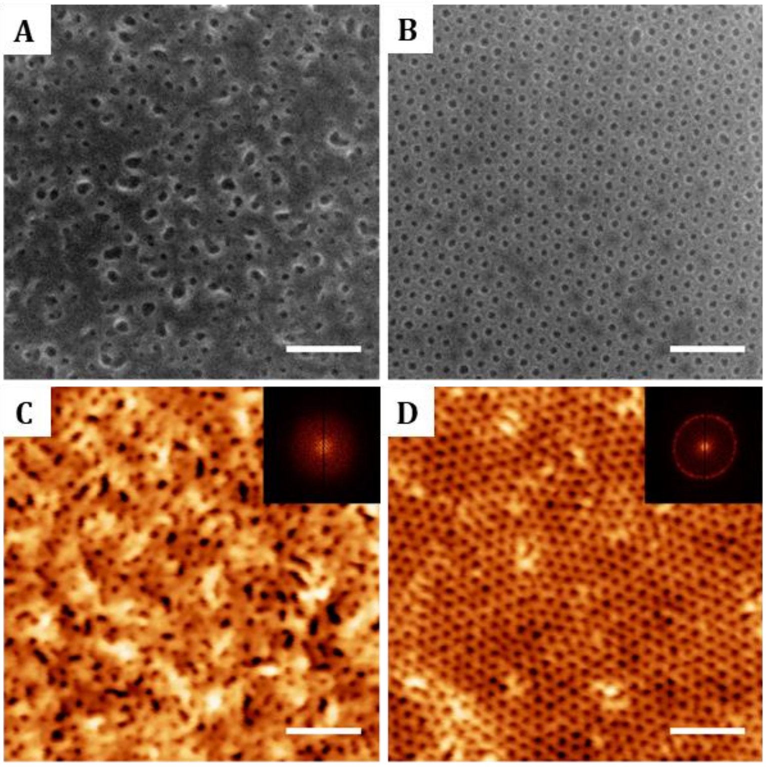

Figure S3. Morphology of PS-b-PMMA 46-21 diblock copolymer thin films after PMMA etching. SEM images of the nanostructured surfaces before (A) and after thermal annealing (B). AFM images before (C) and after annealing (D). Insets shown FFT performed on AFM images. (Scale bar = 400 nm, Z-scale for AFM images = 5 nm).
